# Retrospective Abstract of the Journal

**Published:** 1849

**Authors:** 


### Part 4.—Editorials.

### ARTICLE I.

#### RETROSPECTIVE ABSTRACT OF THE JOURNAL.

The last number closed the first volume of the North-Western Medical and Surgical Journal, being the fifth volume since the work was commenced under another title, by Prof. Blaney.

After closing the volume, we look back over the labors of the year, satisfied that we have made some improvement in the work, though it falls far short of what we could desire, and of what we intend it to be, hereafter, if unwearied application on our part, availing ourselves of the best sources of information, and of the contributions of the best physicians in the country, will make it better.

We must award to our numerous contributors our best thanks, for the ability and liberality with which they have assisted us in our labors; enabling us to present as great a variety of original matter, as any of our cotemporaries, who have testified to the merits of many of the articles by transfering them to their own papers.

By way of review of the original department of the volume, we will proceed to give a brief notice of the several communications in the order of their appearance.

No. I. Article 1.—*Inflammation of the Vena Porta.*—Translated from the German of Waller, by Dr. D. Stahl, of Quincy, Illinois.

In directing the attention of the profession to a pathological condition of grave importance, which might be expected here in the west, where abdominal diseases are common, Dr. Stahl has rendered essential servicc by the translation of this excellent article.

Investigations should be instituted to ascertain the extent of this malady by post mortem evidence. The vena porta is seldom examined.

ARTICLE 2.—*Intestinal Worms—Thier Effects, &c., in the Adult.*—By W. Matthews, M. D., of Eberle, Indiana.

This article points out the obscurity of the symptoms of invermination, showing that diseases dependent upon it are frequently unsuccessfully treated as idiopathic affections.

The production of worms in the hog by eating acorns, in the case referred to, corrobates the well established doctrine of indigestible food being a cause of worms, and may furnish a hint to farmers. The cases reported are interesting and show the importance of a thorough acquaintance with general pathology in the practice of medicine, to enable the physician to steer clear of blunders in diagnosis, and consequently in treatment.

ARTICLE 3.—*Retroversion and Antiversion of the Uterus.*

Being by the writer, this article will not receive further notice here.

No. II. ARTICLE 1.—*Purulent Ophthalmia.*—By E. G. MEEK, M. D., of Indianapolis, Indiana.

This article is highly interesting as corroborating the importance of an early resort to the application of Nitras Argenti, in strong solution, to the conjunctiva, in purulent ophthalmia, and has also a bearing upon the question of its applicability in inflammation of other mucus surfaces which is attracting the attention of the profession generally at this time. The author thinks there can be little doubt of the contagious nature of the disease. It is written in a clear, familiar, and interesting style. It was republished in the Medical Examiner.

ARTICLE 2.—*Medical Literature,* By Prof. FITCH.

The object of this article is to put the profession on the alert in reference to mere assertions and opinions, and to notice the false colouring frequently given by authors to their facts by partial statements and sophistical deductions. It gives a judicious statement of the responsibilities of authors and reviewers in reference to these matters.

ARTICLE 3.---*Old Dislocation of the Humerus.* By H. S. HUBER, M. D., of Chicago.

This was a dislocation downward and forward of thirty-three days standing, which was reduced by the use of the ordinary means except that a twisted rope was used instead of a Pulley.

The favorable result is attributed to the care observed in making the extension, and to the scapula being confined to prevent its being drawn from the body, which the author thinks would endanger the artery by its tension. There is, doubless, value in the suggestion, but that an adhesion between the humerus and artery would not effect a rupture of the latter under even this care, is by no means probable.

ARTICLE 4.—*A Case of Purpurea Hemorrhagica* By W. WADSWORTH, M. D., of Racine, Wisconsin.

This case is valuable as showing the beneficial influence of Spirits Terebinth.; but by no means proves that this will be a specific in hemorrhage as has been suggested by some. That its peculiar stimulant effects are beneficial in the disease in question we have no doubt. The article has been copied by one or two of our exchanges.

ARTICLE 5.---*Death by Vaccination.* By Dr. GREGG, of Rock Island, Illinois.

That erysipelas should follow vaccination is not remarkable, since it sometimes is observed to follow the slightest injuries, but here we have a good cause for the character of the disease in question, as there can be little doubt of the contagion having been contracted previously. It has been copied and noticed, by most of our exchanges.

ARTICLE 6.---*Treatment of Hydrocele.* By L. DUNLAP, M. D., of Indianapolis.

After a brief statement of a few of the authorities in favor of the modes of treatment by injection and incision the author decides in favor of the latter. He advises that the tent inserted be made of a piece of narrow silk ribbon. He thinks the danger of violent inflammation not so great in malarious districts of country.

Prof. Dudley, of Kentucky, has long pursued a practice similar to that recommended, i. e. in the use of the tent.

ARTICLE 7.---*Case of Abnormal position of the Intestines, in the opposite side of the Body.* Translated from the German of Wolshoffer, by Dr. STAHL, of Quincy, Illinois.

This, and the case of Mr. Whitman refered to, are not only interesting as showing the freaks of nature, but they put us on the look out for such cases in examining for diseases of these organs.

ARTICLE 8---*Observations on Cholera Infantum.* By J. H. McNUTT, M. D., of Annapolis, Indiana.

This is an excellent article and makes many valuable practical suggestions in reference to the treatment of this fatal malady. The experience of the author confirms the utility of the following proscription:

℞.---Rad Rheii (burnt);

Loaf Sugar, a a ℥ji;

Water, f℥vii.

Boil for a few moments and strain, to each ounce of the decoction add Bicarb. Soda, grs. v. Dose, a teaspoonful given three or four times daily.

The suggestion that physicians should be careful not to attribute green-colored discharges to hepatic derangement, and make it an indication for the use of calomel, is a good one, and ought to be more generally heeded.

ARTICLE 9.---*Proceedings of Medical Societies.*

Among the most encouraging signs of the times is the disposition we find in the profession to organize. It is the true policy. Argeeably to De Tocqueville, this is the great feature of American policy upon which success depends.

Organize brethren; write papers and read them to your meetings, giving the results of your observations, taking care to investigate thoroughly the subject upon which you write, and you cannot fail to improve. Then when you have read an article that will instruct the members of your associations, remember it may do good if published and send it in.

NO. III. ARTICLE 1.--- *Case of un-united fracture of the Femur, of one years standing, successfully treated by Re-section Denudation, and retaining the ends of bone, by means of wire* By Prof. BRAINARD.

This class of cases has heretofore been treated by amputation, as may be seen by reference to the description. The plan of treatment pursued is original, ingenious, and admirably adapted to fill the indications. The article was copied by the American Journal of Medical Science, in the same number of which is the report of a very similar case treated by one of our most eminent surgeons, which resulted in amputation.

ARTICLE 2.--*Old Dislocation of the Humerus, mistaken for recent injury and consequent failure of the attempt to reduce.* By H. ROSENKRANS, M. D., of Ottawa, Illinois.

Mistakes of this kind when reported have a valuable influence in showing how like errors may be avoided. Physicians are too backward in reporting their failures. A thorough examination of the case no doubt would have revealed its nature; but who when called to a case of dislocation of the shoulder, stops to make a critical examination before attempting a reduction?

ARTICLE 3.—*Strictures on the use of Ergot as a Parturient Agent.* By JNO. H. SANDERS, M. D., of Indianapolis, Ind.

The author condemns, in unqualified terms the use of ergot as an aid in effecting delivery in parturition.

The objections urged are, 1st, the difficulty of determining the cause of obstruction to labor; 2d, the great suffering superinduced; 3d, the contractions induced by it are more like spasms than expulsive efforts; 4th, the great liability of its inducing uterine inflammation; 5th, it frequently causes the death of the child; 6th, liability of injuring the vagina; 7th, a morbid sensibility induced by it; 8th, healthy secretions interrupted by its influence; 9th protracted recovery often follows; 10th, liable to be used, when not necessary, to save time. He has practiced twenty years without finding its use necessary.

Although some of the objections are not very forcible, there are many points presented, bearing strongly against a general use of the article. The influence of the paper upon the profession will be good, if it checks the practice of resorting to the use of ergot unnecessarily to hasten delivery.

ARTICLE 4.---*Observations upon Abdominal Movements as connected with Pregnancy.* By JNO. E. MCGIRR, M. D., of Chicago.

Two cases are reported which show the uncertainty of movements of the abdomen, as a symptom of pregnancy and the author is strong in his praise of the stethoscope as a means of diagnosis in pregnancy.

ARTICLE 5.---*Adipose Tumors.* By JNO. T. PLUMMER, M. D., of Richmond Indiana.

The author's labors in the field of natural science have secured him a high and deserved reputation. The article is interesting and gives some valuable facts bearing upon the nature and pathology of tumors.

ARTICLE 6.---*Singular Case of Uterine Fleshy Mole.* By R. R. STONE, M. D., of Dorr, Illinois.

This is a remarkable case and presents some features of difficult explanation. It has been copied into several of our exchanges.

No. IV. ARTICLE 1.---*Medical Topography of Greencastle, Indiana, and Vicinity.* By R. CURRAN, M. D.

This is a well written statement of the facts, the title purports to give, with philosophical deductions from them, and would triumphantly refute the charge, if made, that Greencastle is an unhealthy situation. As our readers are many of them so situated that opinions may be asked by parents in reference to sending their sons to the University there, the information given here will be of particular utility, and enable them to speak understandingly on the subject.

ARTICLE 2.---*Hernia Cerebri.* By G. W. MEARS, M. D.

This is an account of a disease of rather rare occurrence, upon which but little has been written. The case reported, and the practical suggestions, are instructive and interesting.

ARTICLE 3.---*Abdominal Movements in reference to Pregnancy ---their Nature and Cause.* By JNO. E. MCGIRR, A. M., M.D., etc.

This article is a continuation of the subject from an article already noticed. The conclusion that the child is not the cause of the motions generally attributed to it, we think a mistake; yet they are liable to be similated, and the accoucheur should be on his guard and not place too much reliance upon them. A case came under our observation, in which the patient supposed herself in the fifteenth month of gestation. The motions were so strong that the patient could scarcely sleep at night, and, upon placing the hand upon the scrobiculis cordis, became most violent. It was found that the motions were spasmodic contractions of the diaphragm, partly, at least, under the control of the will.

ARTICLE 4.---*A case of Cerebral Abscess.* By THOS, HALL, M. D., of Toulon, Ill.

This is an interesting case, showing the correctness of symptoms in making out a diagnosis which was confirmed by post mortem demonstration. The members of the profession generally neglect to qualify themselves from making these examinations, which is a great defect in their education, for they should at all times be ready to investigate the post mortem evidences of their correctness or error in in diagnosis. By the way a correction has been made by Dr. Hall in the account of the paralysis, it being on the opposite side from the abscess, as is usually the case, instead of the corresponding side, as reported.

Article 5.---*Posoning by Coculus Indicus.* By H. Rosenkrans, M. D., of Ottawa.

This shows the dangei to the human constitution of an article generally regarded as poisonous of the effects of which but little is known. The rapidity with which it proved fatal shows its virulence. The article has been copied by most of our exchanges.

Article 6.---*New Treatment of some cases of Placenta Prævia.* By H. S. Huber, M. D., of Chicago.

Primal pressure has long been known to excite uterine contractions to some extent, but we rely but little upon it in a case demanding such active treatment as placenta prævia generally does. It, however, can be resorted to while other means of treatment are applied, and might be valuable if Dr. Simpson's plan of separating the placenta from its attachment to the uterus had been resorted to.

Article 7.---*A case of Neuralgia.* By J. George Osborne, M. D., of Tippecanoe county, Indiana.

Tobacco is powerfully narcotic, and is manefestly capable of producing nervous derangement; but as causing neuralgia it is but little known. This is a highly interesting case, and has been copied by a number of our exchanges.

No. V. Article 1.---*Sanguinaria Canadensis as a Therapeutic Agent,* By J. L. Mothershead, M. D., of Indianapolis Indiana.

Although, in the cases reported, in this article, the symptoms are not described as fully as could be desired, valuable practical facts are given showing the use of blood root, especially in dispepsia.

Article 2.---*Remarks on Puerperal Fever complicated with Malaria.* By E. R. Parks, M. D.

This article calls the attention of the profession to a diseased condition of the puerperal state, which most of our readers will remember to have met with, and many of them often, that has heretofore, so far as we recollect to have seen, not been fully described. It is a valuable contribution to our western medical literature.

ARTICLE 3.---*Rheumatic Affections of the Stomach.* By R. R. STONE, M. D., of Solon, Illinois.

This is a sensible article, and makes a valuable practical suggestion in reference to the use of colchicum in the chronic pains "in the sides and shoulders so often complained of in this latitude."

ARTICLE 4.---*Autopsy of a case of Abscess of the Liver.* By H. ROSENKRANS, M. D., of Ottawa, Illinois.

Excepting the encouragement to the practice of making post mortem examinations, this case seems to possess but little interest. It shows the oft proven fact that abscess of the liver is liable to discharge through the lungs.

ARTICLE 5.---*Diseases of the West---their complications, etc* By W. MATHEWS, M. D., of Eberle, Indiana.

This article is a contribution to our stock of facts on "sectional medicine," and shows that the treatment, of some cases at least, of inflammation should be varied from the general rules laid down for its management. It is also shown tha some practical knowledge of western peculiarities is required to detect the complication.

ARTICLE 6.---*A case of Strangulated Hernia mistaken for bilious colic by a Thompsonian---consequent death.* By R. HORSLEY, Medical Student.

This is another instance of the fatal consequences that are continually resulting from the lamentable ignorance of quacks

ARTICLE 7.---*Report of a case of Inflammation of the Fauce ---closure---tracheotomy---recovery.* By J. B. HERRICK, M. D· Demonstrator of Anatomy in Rush Medical College.

This is a well reported case and shows the entire safety o the operation of tracheotomy when skilfully performed. It has been copied by one or two of our cotemporaries.

Article 8.---*Polypus Uteri.* By Dr. R. C. Hamill, of Bloomington, Indiana.

This is a well reported case, treated successfully by ligation.

Article 9.---*Letter from Willis G. Edwards, M. D., to Dr. Brainard.*

This letter gives an interesting account of the surgery of the hospitals of Paris, after the bloody battles of last June.

Article 10.---*Iodine an Antidote to the bite of the Rattle Snake.* By J. Whitmier, M. D., of Metamora, Illinois.

This is an original suggestion and, from the recommendation of the author, deserves further trial. It has been copied or noticed by most of our exchanges.

No. VI. Article I.—*Case of Malignant Pneumonia, with Remarks.* By Edward R. Parks M. D., of Indiana.

The cases of disease reported, and the treatment parsued, illustrate the necessity of guarding well against the endemic influence by which the diseases of the Western country are modified, and show the fatal consequences of an ignorance of them. To the practitioners in the west this is a most valuable document.

Article 2.---*Remedy for Deafness in cases of Destruction of the Tympanum and small bones of the Ear.* By C. Brackett, M. D., of Rochester, Indiana.

That a piece of moist cotton should act as a substitute for the membrana tympani, seems to point to the possibility of introducing a substance that will answer the purpose permanently. Dr. B. deserves credit for the suggestion.

Article 3.---*A case of Malformation of the Heart.* By J. Jackson, M. D. of Goshen, Indiana.

Many cases die of imperfect respiration, some at an early age, others at a later period, but that a simple opening in the foramen ovale would cause death without some other disturbance in the equilibrium between the pulmonary and general circulations, we much doubt. This case is of interest as bearing upon this point. A difficulty in respiration will prevent the closure of the foramen; but a want of closure of the foramen ovale will not produce a difficulty in respiration.

ARTICLE 4.—*Remarks on Belladonna as an Application to Ulcers.* By E. S. COOPER, M. D., of Peoria, Illinois.

The cases referred to here are amongst the most perplexing met with, and the use of an article that promises to cure readily, and relieve from suffering speedily, is valuable. The use of the Belladonna has not been adopted as extensively as it deserves.

ARTICLE 5.—*Influence of Disordered Functions of the Skin in producing Fever.* By J. JACKSON, M. D.

This theory of fever is becoming quite popular and, to say the least of it, quite as likely to prove true as the doctrine of the cryptogamous origin.

The suppression of the perspiration is the arrest of an emunctorial process, which would be expected to produce constitutional derangements.

ARTICLE 6.—*History of a case of Spinal Disease.* By Dr. M. F. IRWIN, of Chrystal Lake, Illinois.

The report of unusual cases, especially when accompanied with full accounts of symptoms and autopsies are valuable contributions to our stock of knowledge of disease.

ARTICLE 7.—*Address to the Graduating class of Rush Medical college.* By D. BRAINARD, M. D., Pres't of the College.

This address is marked by the vigorous style and thought of the author, and presents the claims of our profession in such light that they can but be respected.

ARTICLE 8.—*Treatment of Spina Bifidia by Injections of Tr Iodine.* By Prof. BRAINARD.

This treatment, originally suggested by the author, and its application to large suppurating cavities, is likely to prove a most important discovery in Surgery.

The grateful acknowledgements of ourselves and readers are due to our contributors for articles many of which have uncommon merit. We hope that they will continue to labor with us in the field of science. E.

